# Integrating ultrasound and clinical risk factors to predict carotid plaque vulnerability in gout patients: a machine learning approach

**DOI:** 10.3389/fmed.2025.1556387

**Published:** 2025-06-19

**Authors:** Yabin Fang, Kaiyi Yang, Xinyu Gao, Yiran Gong, Yaxin Deng, Xiang Xu, Jing Xu, Lei Yan, Jinshu Zeng, Shuqiang Chen

**Affiliations:** ^1^Department of Ultrasound, The First Affiliated Hospital of Fujian Medical University, Fuzhou, Fujian, China; ^2^Department of Ultrasound, National Regional Medical Center, First Affiliated Hospital of Fujian Medical University Binhai Campus, Fuzhou, Fujian, China; ^3^Department of Ultrasound, Fuzhou University Affiliated Provincial Hospital, Fuzhou, Fujian, China

**Keywords:** gout, risk stratification, ultrasound, carotid plaque, inflammation, prediction model, diagnosis

## Abstract

**Objectives:**

This study aimed to identify independent risk factors for carotid plaque (CP) vulnerability in patients with gout and to develop a predictive model incorporating both gout-specific and cardiovascular factors.

**Method:**

This study was designed as a retrospective cohort analysis that enrolled patients with newly diagnosed gout. These patients were retrospectively followed for a period of 1 to 2 years to evaluate the incidence of CP vulnerability. CP vulnerability was assessed using standardized ultrasound examinations and graded according to the Plaque Reporting and Data System (Plaque-RADS). Multivariate ordinal logistic regression analysis was employed to identify independent risk factors associated with CP vulnerability, with a particular focus on the impact of gout-related variables. Based on these results, a random forest prediction model was developed by integrating ultrasound imaging features and clinical variables to predict CP vulnerability.

**Results:**

Tophi (OR = 1.760, *p* = 0.009), power Doppler (PD) signal grades (Grade 2: OR = 1.540, *p* = 0.002; Grade 3: OR = 1.890, *p* = 0.001), and the number of gout flares in the last year (OR = 1.524, *p* = 0.001) were identified as independent risk factors for CP vulnerability. The random forest model showed excellent predictive performance (C-index = 0.997) and highlighted tophi, PD signal grades, and gout flare frequency as key gout-specific contributors to CP risk.

**Conclusion:**

The presence of tophi, positive PD signals, and increased number of gout flares are significantly associated with CP vulnerability in patients with gout. The proposed machine learning model, integrating gout-specific and cardiovascular factors, provides a novel and effective approach for personalized risk stratification and management in gout patients, bridging the gap between rheumatic inflammation and cardiovascular risk assessment.

## 1 Introduction

Gout is a chronic metabolic disease defined by the accumulation of monosodium urate (MSU) crystals in joints, tendons, and surrounding tissues (1). The reported prevalence of carotid atherosclerosis in patients with gout ranges from approximately 29.1% to 48.9% (2) with nearly half showing carotid plaques (CPs) on ultrasound imaging (3). While increases in intima-media thickness and CP formation are important markers of carotid atherosclerosis, a more clinically relevant concern is plaque vulnerability, which serves as a critical predictor of cerebrovascular events, including stroke (4).

Recent studies suggest that gout contributes to carotid atherosclerosis not only via hyperuricemia but also through sustained low-grade inflammation induced by MSU crystals (5). These crystals stimulate the activation of interleukin-1β (IL-1β) and neutrophil extracellular traps (NETs), thereby exacerbating oxidative stress and endothelial dysfunction (6, 7). Despite the established link between gout and CP development, studies investigating the associations of urate crystal deposition and inflammatory markers with CP vulnerability in patients with gout remain limited.

Compared to magnetic resonance imaging (MRI) and dual-energy computed tomography (DECT), ultrasound offers notable advantages in the early diagnosis and monitoring of acute gout, particularly in detecting minute urate crystal deposits and those on cartilage surfaces (8). As a result, it has been incorporated into the latest classification criteria for gout, as outlined by the American College of Rheumatology and the European Alliance of Associations for Rheumatology (ACR/EULAR) guidelines (9). In addition, gout-specific features related to urate crystal deposition—such as the double contour sign (DCS), hyperechoic aggregates (HAG), and tophi—have been identified, facilitating the quantification of joint damage severity (10, 11).

In this study, we utilized ultrasound to evaluate joint lesions in patients with gout and explored the feasibility of developing a predictive model that integrates ultrasound and clinical features to assess CP vulnerability. This approach aims to elucidate the complex relationship between gout and cardiovascular as well as cerebrovascular events, offering a novel perspective for risk stratification.

## 2 Materials and methods

### 2.1 Patients

**FIGURE 1:**
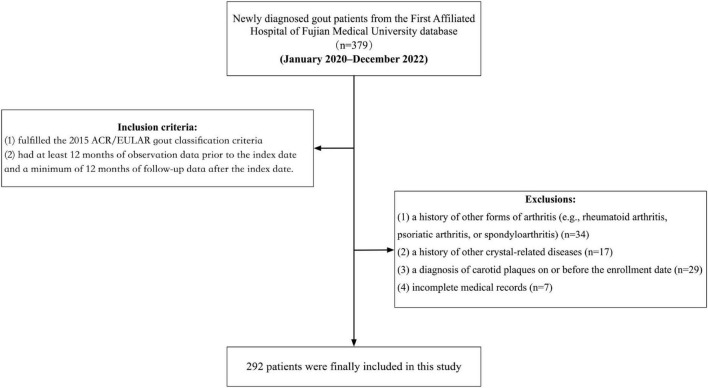
Flowchart of patients.

This retrospective cohort study was based on the database of the First Affiliated Hospital of Fujian Medical University, comprising anonymized data on medication prescriptions, diagnoses, basic clinical records, and demographic characteristics, directly retrieved from the hospital’s system. The study period spanned from January 2020 to December 2022, during which participants were consecutively enrolled based on newly diagnosed cases of gout. Eligible participants were those who met the following inclusion criteria at the time of diagnosis: (1) fulfilled the 2015 ACR/EULAR gout classification criteria; (2) had at least 12 months of observation data prior to the index date and a minimum of 12 months of follow-up data after the index date. The exclusion criteria were: (1) a history of other forms of arthritis (e.g., rheumatoid arthritis, psoriatic arthritis, or spondyloarthritis); (2) a history of other crystal-related diseases; (3) a diagnosis of carotid plaques on or before the enrollment date; and (4) incomplete medical records. After applying these stringent inclusion and exclusion criteria, a total of 292 adult patients with an initial diagnosis of gout were included in the study (Figure 1).

This study was in accordance with the Ethical Standards of the Institutional Ethics Committee of First Affiliated Hospital of Fujian Medical University and with the 1964 Helsinki declaration and its later amendments or comparable Ethical Standards. Ethics batch number: MRCTA, ECFAH of FMU [2022]251. As a purely retrospective review of medical records that did not involve any personally identifiable information, the requirement for informed consent was waived.

### 2.2 Variables

#### 2.2.1 Outcome variable

**FIGURE 2:**
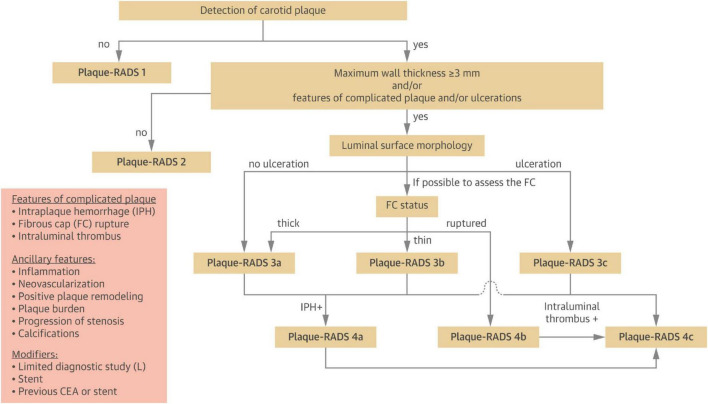
Step-by-step flow chart to classify carotid plaques into the different Plaque-RADS categories (13).

**FIGURE 3:**
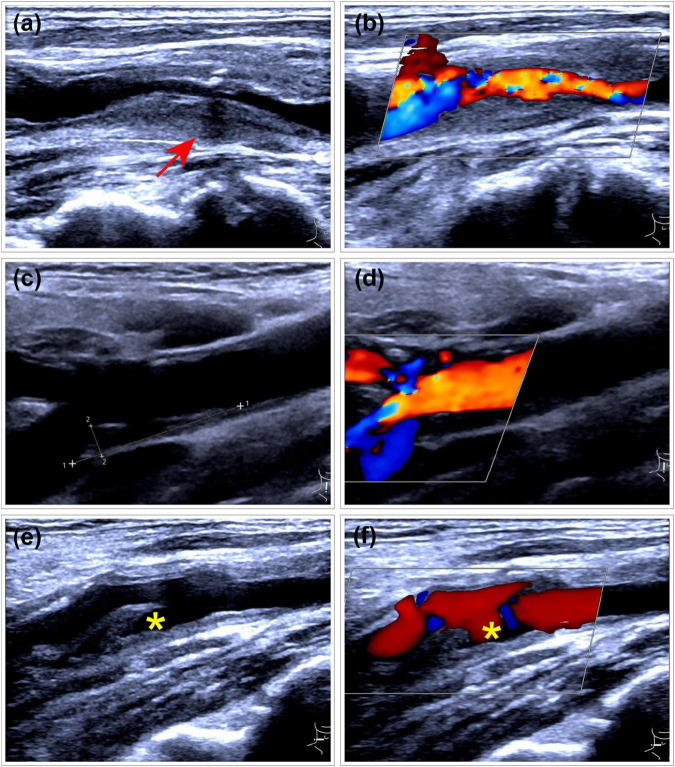
Representative ultrasound images of Plaque-RADS Category 3 subtypes (3A–3C). **(a,b)** RADS 3A plaque at the carotid bifurcation with a maximum wall thickness (MWT) greater than 3 mm. The plaque exhibits a uniform isoechoic appearance (arrow) and features a thick fibrous cap. **(a)** Gray-scale ultrasound imaging. **(b)** Doppler flow imaging. **(c,d)** RADS 3B plaque at the carotid bifurcation with a MWT greater than 3 mm. The plaque contains multiple very low echogenic areas, with most regions lacking a visible (thin) fibrous cap. **(c)** Gray-scale ultrasound imaging. **(d)** Doppler flow imaging. **(e,f)** RADS 3C plaque with a mixed hyperechoic and hypoechoic plaque at the carotid bifurcation, demonstrating ulceration (*) on both two-dimensional and Doppler imaging.

The outcome variable in this study was CP vulnerability assessed by ultrasonography. Carotid ultrasound assessments were performed using images acquired during examinations conducted 1–2 years after the diagnosis of gout. Atherosclerotic plaques were identified bilaterally in the common carotid artery (CCA), carotid bulb, and internal carotid artery (ICA). The presence of CPs was determined based on the Mannheim consensus (12), with only plaques exhibiting well-defined contours included. Plaque vulnerability was assessed using the Plaque-RADS classification system proposed by Saba et al. (13), and the complete criteria are presented in Figure 2. Figure 3 illustrates representative ultrasound characteristics of category 3 subtypes (3A–3C). For patients with multiple CPs, the plaque with the highest Plaque-RADS score was selected for analysis.

#### 2.2.2 Explanatory variable

Explanatory variables were selected based on a preliminary case-control study that included a carotid plaque group (case group) and a plaque-free group (control group), matched for age and sex. A LASSO regression model based on L1 regularization was employed to analyze all candidate independent variables. Through this process, 17 non-zero coefficient variables associated with plaque formation were identified, while smoking and alcohol consumption were excluded. These variables were subsequently included as independent variables in the retrospective cohort study (Details of the pilot study are provided in the Supplementary Data 1).

**FIGURE 4:**
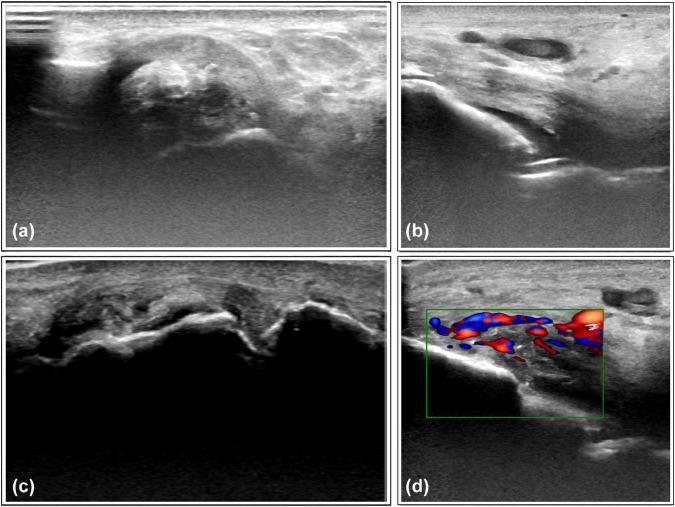
Representative ultrasound features of monosodium urate crystal deposits and associated inflammation. **(a)** Tophi observed on the lateral aspect of the ankle joint. **(b)** Double-track sign on the medial side of the ankle joint. **(c)** Scattered hyperechoic aggregates with bone erosion observed in the 1st metatarsophalangeal joint. **(d)** Intra-articular blood flow signals detected in the ankle joint capsule, suggesting synovitis.

The primary explanatory variables in this study focus on ultrasound findings and clinical characteristics related to gout. Musculoskeletal ultrasound assessments were conducted during the intercritical period, within 30 days of gout diagnosis, adhering to the standardized protocols outlined by the Outcome Measures in Rheumatology (OMERACT) working group (10). Ultrasound images of the first metatarsophalangeal joint (MTP1), ankle, and knee joints were re-evaluated for each patient. At the anatomical level, the assessment included: (a) key lesions of MSU crystal deposition, including the DCS, HAG, and tophi (11) (Figure 4) using a binary scoring system (presence/absence) as recommended by Naredo et al. (14). (b) inflammatory markers, assessed through the presence of local PD signals and bone erosion, graded using a semi-quantitative 0–3 scoring system (15) (Figure 4). Additionally, clinical features of gout were included, including the number of affected joints, the number of gout attacks in the last year, and the duration of the disease.

Secondary explanatory variables encompassed demographic and clinical characteristics, including serological test results, cardiovascular risk factors, comorbidities, and treatment regimens (Full list of explanatory variables provided in the Supplementary Data 2).

All ultrasound assessments were conducted with a standardized device model (SAMSUNG RS9 system) utilizing a high-frequency linear transducer (6–14 MHz). Stored ultrasound images were independently and blindly evaluated by two experienced sonographers, each with over 10 years of clinical practice, to ensure data reliability.

### 2.3 Statistical analysis

Following assessment of the normality of continuous variables and the homogeneity of variances, data were reported as means with standard deviations (SD) or medians with interquartile ranges (IQR). Categorical variables were presented as frequencies (*n*) and percentages (%).

Multivariable ordinal logistic regression combined with a stepwise backward elimination method was employed to identify independent risk factors associated with CP vulnerability. In addition, univariable logistic regression analyses were performed for smoking and alcohol consumption to inform subsequent sensitivity analyses. To evaluate the incremental predictive value of gout-specific factors, a conventional cardiovascular risk model was constructed by omitting these variables from the final model. The performance of both models was subsequently compared using cumulative receiver operating characteristic (ROC) curve analysis. The identified risk factors were subsequently used to construct a Random Forest prediction model, with variable importance assessed by the Mean Decrease in Gini Index.

Model adequacy and stability were assessed via the Akaike information criterion (AIC) and Bayesian information criterion (BIC), McFaddens’ pseudo-R^2^ and concordance index (C-index). Although smoking and alcohol consumption were excluded by LASSO selection due to limited statistical contribution, they were included in a sensitivity analysis given their clinical relevance as cardiovascular risk factors and potential confounders (16). Model robustness was assessed by evaluating changes in key effect estimates of primary risk factors following their inclusion. Performance evaluation of the random forest model includes Accuracy, Precision, and Recall from the confusion matrix, and the goodness-of-fit of the model is assessed using the Mean Absolute Error (MAE).

All statistical analyses were conducted using R (version 4.1.1), with a two-sided *p*-value < 0.05 considered statistically significant. All participants with complete data were included in the analyses; i. e. complete case analysis.

## 3 Results

### 3.1 Clinical, demographic, and gout characteristics

A total of 292 initially diagnosed gout patients were included in the study between January 2020 and December 2022. Table 1 presents the characteristics of the enrolled participants. The cohort consisted predominantly of male patients, with a median age of 61 years. During follow-up, 167 cases (57.2%) were found to have CP. According to the Plaque-RADS classification system, 42.8% were classified as category 1, 32.2% as category 2, and the remainder were distributed across higher categories. Notably, no plaques were classified as category 4. Among the patients with CP, 56 (19.2%) experienced cerebrovascular events, such as stroke or transient ischemic attack.

**TABLE 1:** Population, clinical, and ultrasound characteristics in a gout cohort.

Characteristics	Overall (*N* = 292)
Age in years, median (IQR)	61.0 (51.0, 69.0)
Men, ***n*** (%)	245 (83.9)
BMI (kg/m^2^), median (IQR)	25.0 (23.2, 26.7)
Uric acid (umol/L), median (IQR)	401.0 (359.0, 486.8)
Cholesterol (mmol/L), median (IQR)	4.4 (3.4, 5.4)
LDL (mmol/L), mean (SD)	2.7 (1.2)
CKD (GFR < 60 mL/min), ***n*** (%)	32 (11.0)
Diabetes, ***n*** (%)	112 (38.4)
Coronary heart disease, ***n*** (%)	49 (16.8)
Cerebrovascular disease, ***n*** (%)	56 (19.2)
Antiplatelet drugs, ***n*** (%)	83 (28.4)
Urate-lowering therapy, ***n*** (%)	193 (66.1)
Antihypertensive drugs, ***n*** (%)	178 (61.0)
**Charlson index, n (%)**	
0	20 (6.8)
1–2	128 (43.8)
3–5	103 (35.3)
≥ 6	41 (14.0)
**Gout-related disease characteristics**	
Number of flares last year, median (IQR)	3.0 (2.0, 5.0)
Number of involved joints, median (IQR)	3.0 (1.0, 4.0)
Disease duration, median (IQR)	5.0 (1.0, 7.0)
**MSK-US findings in Gout**	
Gout tophi, ***n*** (%)	148 (50.7)
DCS, ***n*** (%)	196 (66.9)
HAG, ***n*** (%)	159 (54.3)
**PD signal, n (%)**	
0	131 (44.9)
1	92 (31.5)
2	42 (14.4)
3	27 (9.2)
**Bone erosion, n (%)**	
0	97 (33.2)
1	108 (37.0)
2	55 (18.8)
3	32 (11.0)
cPP, ***n*** (%)	167 (57.2)
**Plaque-RADS, n (%)**	
1	125 (42.8)
2	94 (32.2)
3a	45 (15.4)
3b	24 (8.2)
3c	4 (1.4)

BMI, body mass index; IQR, interquartile range; LDL, low-density lipoprotein; SD, standard deviation; CKD, chronic kidney disease; GFR, glomerular filtration rate; MSK-US, musculoskeletal ultrasound; DCS, double contour sign; HAG, hyperechoic aggregates; PD, power Doppler; cPP, carotid plaque presence; RADS, Reporting and Data System.

Regarding the clinical characteristics of gout, the time from the first flare to diagnosis was notably prolonged (median 5 years). The number of gout flares in the year prior to diagnosis was relatively high (median 3 episodes). Additionally, 148 patients (50.7%) presented with tophi. In terms of musculoskeletal ultrasound findings, more than half of the patients exhibited evidence of the DCS (66.9%) or HAG (54.3%). PD signals were detected in 55.1% of patients, with grades 2–3 observed in 23.6%. Bone erosion was identified in 66.8% of the cases.

### 3.2 Association between gout and CP vulnerability

**FIGURE 5:**
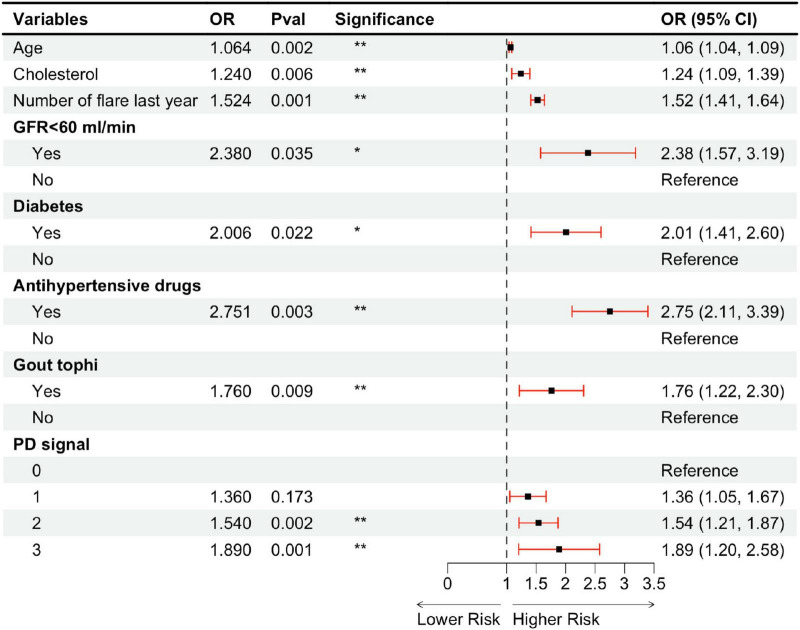
Multivariable ordinal logistic regression model assessing carotid artery plaque vulnerability in a gout cohort. OR, odds ratio; CI, confidence interval; GFR, glomerular filtration rate; PD, power Doppler. *P < 0.05; **P < 0.01.

Using a LASSO regression model with L1 regularization, 17 variables with non-zero coefficients were identified. These variables were subsequently included in a multivariate logistic regression analysis employing stepwise backward elimination, which yielded a final model comprising eight variables: age, cholesterol level, number of flares in the last year, impaired renal function (GFR < 60 ml/min), diabetes, use of antihypertensive medications, the presence of gout tophi, and PD signal grade (Figure 5). Among ultrasound features, the presence of tophi (OR = 1.760, *p* = 0.009) and the degree of positive PD signals (grade 2: OR = 1.540, *p* = 0.002; grade 3: OR = 1.890, *p* = 0.001) were identified as independent risk factors of higher Plaque-RADS categories, while DCS, HAG, and bone erosion were excluded due to a lack of statistical significance (*p* > 0.05). Regarding clinical characteristics, only the number of gout flares in the last year (OR = 1.524, *p* = 0.001) was a significant risk factors, while disease duration and the number of affected joints were excluded (*p* > 0.05). Specifically, the presence of tophi increased the likelihood of plaque vulnerability by 24%, while positive PD signals were associated with a 54% increase for grade 2 and an 89% increase for grade 3. Additionally, each extra gout flare per year was linked to a 52% higher risk.

**FIGURE 6:**
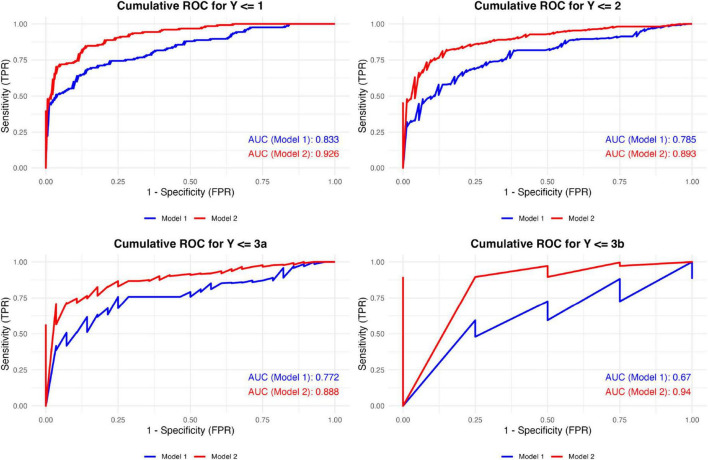
Cumulative ROC curves of the two models across different classification thresholds. Model 1 included traditional cardiovascular risk factors: age, cholesterol levels, GFR < 60 mL/min, diabetes, and antihypertensive medication use. Model 2 extended Model 1 by integrating gout-specific factors, including the presence of tophi, Doppler flow, and the number of gout flares in the last year. ROC, receiver operating characteristic; TPR, true positive rate; FPR, false positive rate.

When evaluating diagnostic performance, the final model—including gout-related variables—consistently outperformed the baseline cardiovascular model across all cumulative binary classification thresholds. The mean AUC values were 0.765 for the baseline model and 0.912 for the final model, demonstrating a substantial improvement in discriminative ability (Figure 6). The final model showed strong performance, with an AIC of 539.69, BIC of 591.16, McFadden’s pseudo-R^2^ of 0.60, and a C-index of 0.88, indicating good model fit and discrimination (see Supplementary Table 1). The univariable results for smoking and alcohol consumption are summarized in Supplementary Table 2. Sensitivity analysis results indicated that the four models exhibited similar effect sizes (β), confidence intervals, and statistical significance across eight common variables, supporting the robustness of the main findings (see Supplementary Data 3).

### 3.3 Random forest prediction model for CP vulnerability in gout patients

A random forest prediction model for CP vulnerability was constructed using independent risk factors identified through multivariable logistic regression analysis. The model exhibited exceptional goodness-of-fit (MAE = 0.096) and discriminative performance (C-index = 0.997). The confusion matrix results further confirmed the model’s high predictive accuracy and strong generalizability in predicting carotid Plaque-RADS categories. Performance metrics were as follows: accuracy = 0.925, precision = 0.960, and recall = 0.905. Details of the confusion matrix for the random forest model are provided in Supplementary Table 3.

**FIGURE 7:**
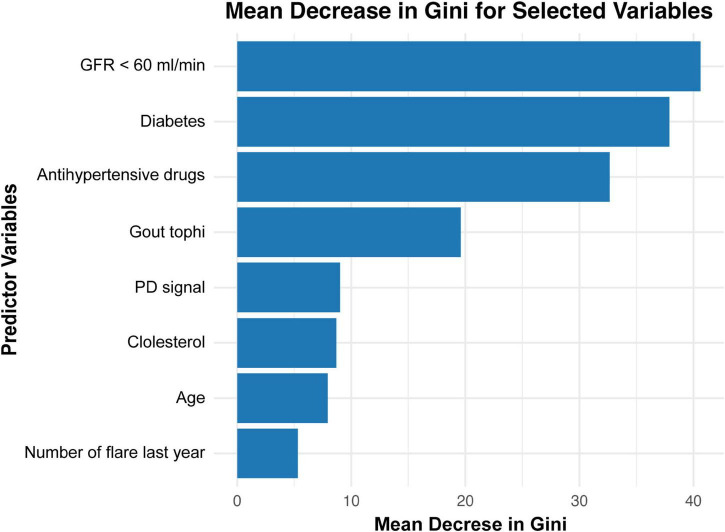
Mean decrease in Gini for feature importance in a random forest model predicting carotid plaque. GFR, glomerular filtration rate; PD, power Doppler.

Variable importance in the decision tree ensemble was evaluated using the Mean Decrease in Gini index. As illustrated in Figure 7, while conventional cardiovascular risk factors—such as impaired renal function, diabetes, and antihypertensive medication use—were among the top predictors of plaque grades, gout-related factors—including musculoskeletal ultrasound findings (presence of tophi, Doppler signals) and the number of gout flares in the last year—also contributed substantially to the prediction of carotid Plaque-RADS categories.

## 4 Discussion

Gout is a chronic metabolic disorder resulting from urate overload and characterized by the deposition of MSU crystals. Accumulating evidence suggests that gout may contribute to increased atherosclerotic burden, which in turn predisposes individuals to a higher risk of cerebrovascular events (17). Traditionally, the assessment of plaque vulnerability has relied on ultrasound-based grading of plaque echogenicity. However, this method is highly subjective and susceptible to operator experience, resulting in limited reliability and consistency across evaluations. The Plaque-RADS classification system, a novel stroke risk stratification framework, offers a more standardized and quantitative approach to evaluating plaque echogenicity, thereby enhancing the objectivity and reproducibility of plaque classification.

Our study focused on carotid plaque vulnerability in patients with gout, employing the Plaque-RADS classification system as the evaluation tool. A predictive model was developed to systematically assess plaque vulnerability based on gout-specific clinical and ultrasound features. The results demonstrated significant associations between CP vulnerability and several gout-related indicators, including the presence of tophi, positive PD signals, and the number of gout flares in the last year. Furthermore, in the random forest model analysis, gout-related variables also played a substantial role in predicting plaque vulnerability. These findings underscore the potential value of incorporating gout-specific characteristics into cerebrovascular risk assessment and management strategies.

The presence of tophi in gout patients indicates advanced disease progression and a high uric acid burden, both of which are characteristic of a chronic inflammatory state that may promote plaque vulnerability (18). Previous studies have demonstrated a significant association between large tophi (> 2 mm) and the presence of carotid plaques (5), and longitudinal cohort data have identified tophi as an independent risk factors of adverse cardiovascular events, such as myocardial infarction and stroke (HR 2.12–5.25, *p* < 0.05) (19). Our study builds upon these findings by establishing a direct link between tophi and CP vulnerability, rather than merely plaque presence. Mechanistically, activation of the NLRP3 inflammasome by tophi induces neutrophil-driven acute inflammation, a process strongly implicated in atherosclerotic plaque vulnerability and rupture (20, 21). Within vulnerable plaques, activated inflammatory cells upregulate matrix-degrading enzymes (e.g., matrix metalloproteinases) and enhance oxidative stress, leading to extracellular matrix degradation and plaque destabilization (22).

The random forest model further emphasized the importance of tophi, ranking it as the top gout-specific risk factors of CP vulnerability and fourth overall among all risk factors, following traditional cardiovascular risk factors including GFR < 60 ml/min, diabetes, and antihypertensive medication use. These findings highlight the critical role of tophi in cardiovascular risk and underscore the need for personalized risk stratification and targeted management strategies in gout patients, particularly those with visible tophi.

Interestingly, no significant association was observed between CP vulnerability and the ultrasonographic features of DCS or HAG, which represent distinct types of MSU crystal deposition. This divergence may suggest that the chronicity of MSU crystal deposition plays different roles in carotid atherosclerosis progression (2, 19). HAG, for instance, may be more indicative of early vascular remodeling, as supported by a previous study that have linked it to increased carotid intima-media thickness (5). Future longitudinal studies in gout patients are needed to explore the dynamic contributions of various crystal types in the development and progression of CP.

Doppler technology enables the assessment of vascularization and blood flow at specific anatomical sites. In chronic inflammatory arthritis, PD signals are widely recognized as markers of inflammation and have been linked to histopathological synovitis (23). During acute gout flares, PD signal intensity is significantly elevated. Notably, persistent PD signals may also be observed during the intercritical phase, often in conjunction with progressive bone erosion (24). As a result, PD signals are considered reliable surrogate markers of crystal-mediated inflammation. Our study revealed a strong association between PD signal intensity and CP vulnerability in gout patients. Specifically, compared to PD grade 0, PD grade 3 was associated with an 89% increase in the probability of an upgraded Plaque-RADS score for CP, while PD grade 2 showed a 54% increase in vulnerability. These findings underscore the value of PD signal intensity as a novel and non-invasive indicator for evaluating cardiovascular risk in gout patients. Moreover, they highlight its potential as a therapeutic target for controlling crystal-mediated inflammation to mitigate atherosclerotic risk.

Ultrasound examination is instrumental not only for diagnosing gout but also for monitoring disease progression. Notably, ultrasound evaluation of the reduction in urate deposition can be used to predict the risk of flare recurrence after discontinuing colchicine (25). Leveraging these capabilities, our study demonstrates that ultrasound biomarkers such as tophi and PD signals are crucial in predicting CP vulnerability. By integrating these ultrasound features with clinical risk factors, our random forest prediction model achieved excellent predictive accuracy and reliability. This highlights the potential of ultrasound biomarkers to guide personalized treatment strategies, enabling more precise and effective management of gout patients with heightened cardiovascular risk.

The number of gout flares is another significant marker of systemic inflammation and uric acid-related metabolic dysfunction. Each flare represents an acute inflammatory response driven by neutrophils and pro-inflammatory cytokines, which not only aggravate local damage but also contribute to systemic endothelial dysfunction and oxidative stress (20, 26). Our study found that each additional gout flare in the preceding year was associated with a 52% increase in the likelihood of an upgraded RADS score in CP. Furthermore, the random forest model ranked the number of gout flares in the last year as a key predictor of CP vulnerability, emphasizing its contribution to the overall model performance. These findings reinforce the importance of optimizing urate-lowering therapy and implementing effective anti-inflammatory interventions to mitigate flare frequency and reduce systemic inflammatory burden. Sustained management of these factors has the potential to improve plaque stability and reduce long-term cardiovascular risks in patients with gout.

### 4.1 Limitations and future directions

Although this study provides valuable insights into the relationship between gout and CP vulnerability, its retrospective design presents limitations that may weaken the ability to infer causality. Secondly, Certain lifestyle variables, such as smoking and alcohol consumption, were recorded in a binary format (yes/no), without further granularity to capture frequency, intensity, or recency. This coarse classification of exposure variables may have increased the risk of residual confounding. Future studies could mitigate potential bias arising from oversimplified exposure grouping by employing more refined data collection methods. Finally, although internal cross-validation was applied to mitigate overfitting, the high C-index of the random forest model suggests potential model overtraining. Future external validation in independent gout cohorts would be valuable to further assess the model’s robustness and generalizability.

## 5 Conclusion

This study confirms that the presence of tophi, positive PD signals, and an increased number of gout flares in the last year are significantly associated with carotid plaque vulnerability in patients with gout. Building on this, we constructed a predictive model that incorporates these gout-specific markers alongside traditional cardiovascular risk factors. The model exhibits high accuracy and reliability, providing a valuable clinical instrument for individualized risk stratification and the management of cerebrovascular events in gout patients.

## Data Availability

The original contributions presented in this study are included in this article/Supplementary material, further inquiries can be directed to the corresponding author.
