# Supplementary material for: Integrating ultrasound and clinical risk factors to predict carotid plaque vulnerability in gout patients: a machine learning approach
Source: Front Med (Lausanne). 2025 Jun 19;12:1556387. doi: 10.3389/fmed.2025.1556387 (PMC12224871; doi:10.3389/fmed.2025.1556387)
Supplement: Supplementary file 1 [file Table_1.docx]

Supplementary Material

# Supplementary Tables

# Supplementary Table 1. The stability and goodness-of-fit of the logistic regression model

| **Model** | **AIC** | **BIC** | **pseudo R-squared** | | **C-index** | |
| --- | --- | --- | --- | --- | --- | --- |
| Model 1 | 644.09 | 677.19 | 0.37 | 0.78 | |  |
| Model 2 | 619.13 | 655.90 | 0.43 | 0.80 | |  |
| Model 3 | 602.83 | 646.95 | 0.48 | 0.83 | |  |
| Model 4 | 592.95 | 640.75 | 0.50 | 0.84 | |  |
| Model 5 | 626.53 | 678.00 | 0.43 | 0.81 | |  |
| Model 6 | 570.22 | 606.99 | 0.53 | 0.86 | |  |
| Model 7 | 539.69 | 591.16 | 0.60 | 0.88 | |  |

Model 1 includes the variables age, cholesterol, CKD, diabetes, and antihypertensive drugs.
Model 2 includes the variables age, cholesterol, CKD, diabetes, antihypertensive drugs, and gout tophi.
Model 3 includes the variables age, cholesterol, CKD, diabetes, antihypertensive drugs, and Doppler flow.
Model 4 includes the variables age, cholesterol, CKD, diabetes, antihypertensive drugs, gout tophi, and Doppler flow.

Model 5 includes the variables age, cholesterol, CKD, diabetes, antihypertensive drugs, double track sign, aggregates, and bone erosion.
Model 6 includes the variables age, cholesterol, CKD, diabetes, antihypertensive drugs, and number of flares last year.
Model 7 the final model, includes the variables age, cholesterol, CKD, diabetes, antihypertensive drugs, gout tophi, Doppler flow, and number of flares last year.
