# Supplementary material for: Integrating ultrasound and clinical risk factors to predict carotid plaque vulnerability in gout patients: a machine learning approach
Source: Front Med (Lausanne). 2025 Jun 19;12:1556387. doi: 10.3389/fmed.2025.1556387 (PMC12224871; doi:10.3389/fmed.2025.1556387)
Supplement: Supplementary file 2 [file Table_2.docx]

Supplementary Material

# Supplementary Tables

# Supplementary Table 2. The univariable results for smoking and alcohol consumption

| **Variable** | **OR** | **95%CI** | | **P-value** | |
| --- | --- | --- | --- | --- | --- |
| Smoking (yes) | 1.045 | 0.658-1.653 | 0.850 | |  |
| Alcohol consumption (yes) | 0.683 | 0.429-1.077 | 0.103 | |  |
