# Supplementary material for: Integrating ultrasound and clinical risk factors to predict carotid plaque vulnerability in gout patients: a machine learning approach
Source: Front Med (Lausanne). 2025 Jun 19;12:1556387. doi: 10.3389/fmed.2025.1556387 (PMC12224871; doi:10.3389/fmed.2025.1556387)
Supplement: Supplementary file 3 [file Table_3.docx]

Supplementary Material

# Supplementary Tables

# Supplementary Table 3. Confusion matrix for the random forest model

| Prediction | Reference | Frequency |
| --- | --- | --- |
| 1 | 1 | 122 |
| 2 | 1 | 3 |
| 3a | 1 | 0 |
| 3b | 1 | 0 |
| 3c | 1 | 0 |
| 1 | 2 | 4 |
| 2 | 2 | 90 |
| 3a | 2 | 0 |
| 3b | 2 | 0 |
| 3c | 2 | 0 |
| 1 | 3a | 1 |
| 2 | 3a | 10 |
| 3a | 3a | 34 |
| 3b | 3a | 0 |
| 3c | 3a | 0 |
| 1 | 3b | 1 |
| 2 | 3b | 3 |
| 3a | 3b | 0 |
| 3b | 3b | 20 |
| 3c | 3b | 0 |
| 1 | 3c | 0 |
| 2 | 3c | 0 |
| 3a | 3c | 0 |
| 3b | 3c | 0 |
| 3c | 3c | 4 |
