# Supplementary material for: Integrating ultrasound and clinical risk factors to predict carotid plaque vulnerability in gout patients: a machine learning approach
Source: Front Med (Lausanne). 2025 Jun 19;12:1556387. doi: 10.3389/fmed.2025.1556387 (PMC12224871; doi:10.3389/fmed.2025.1556387)
Supplement: Supplementary file 4 [file Data_Sheet_1.docx]

Supplementary Material

# Supplementary Data 1

# A preliminary study on independent risk factors associated with carotid plaque formation

**1 Materials and Methods**

**1.1 Study Design and Population**

This retrospective case-control study utilized the database of the First Affiliated Hospital of Fujian Medical University, containing anonymized data on medication prescriptions, diagnostic records, basic medical information, and demographic characteristics. All data were directly retrieved from the hospital system in a de-identified format. The study population included adult patients (aged ≥18 years) who underwent carotid ultrasonography at our hospital between January 2020 and December 2022. Eligible patients were consecutively sampled, applying the following exclusion criteria:(1) Presence of other confirmed sources of thromboembolism (e.g., vasculitis, atrial fibrillation, intracranial embolism); (2) History of neck radiation therapy or ipsilateral carotid surgery/stenting; (3) Coexisting severe diseases that could significantly affect the risk of cerebrovascular events (e.g., severe heart disease, advanced cancer). (4) From the screened population, the case group consisted of consecutively sampled patients with carotid plaques. The control group was composed of randomly selected patients without carotid plaques, matched to cases in a 1:1 ratio based on age and sex.

This study was approved by the Ethics Committee of the First Affiliated Hospital of Fujian Medical University (Ethics Approval No.: [2021]251). Given the retrospective nature of the study and the absence of identifiable personal information, informed consent was waived.

**1.2 Variables**

Based on existing evidence related to atherosclerosis, structured clinical data were collected with consideration of the following independent variables: demographic and clinical characteristics, including laboratory test results, cardiovascular risk factors, comorbidities, and therapeutic medications (a comprehensive list of secondary explanatory variables is provided in Supplementary Material 2).

**1.3 Assessment of Carotid Ultrasonography Findings**

The evaluation of plaque images followed the Mannheim Consensus ^[2]^ to assess the presence of atherosclerotic plaques. Atherosclerotic plaques were defined as localized thickenings or protrusions resulting in arterial lumen narrowing of at least 0.5 mm, disruption of the double-line pattern of intima-media thickness (IMT) parallel to 50% of the adjacent IMT, or a thickness exceeding 1.5 mm from the media-adventitia interface to the intima-lumen interface. ^[3]^

**1.4 Statistical analysis**

After assessing the normality of continuous variables, descriptive statistics were presented as mean (standard deviation) or median (interquartile range). Categorical variables were expressed as frequencies (n) and percentages (%). Group comparisons of continuous variables were conducted using the independent samples t-test for normally distributed data or the Mann-Whitney U test for non-normal distributions. For categorical variables, comparisons were performed using the chi-square test or Fisher’s exact test, as appropriate. To identify significant independent variables, a LASSO regression model was employed to analyze all candidate variables. All statistical tests were two-sided, with a significance threshold set at p ≤ 0.05.

**2 Results**

**2.1 Population and Clinical Characteristics**

A total of 818 participants with complete carotid ultrasound evaluation data were included in this study between January 2020 and December 2022. Participants were evenly divided into two groups through 1:1 age- and gender-matching: patients with carotid plaques presence (cPP, n=409) and those without carotid plaques absence (cPA, n=409). C-reactive protein (CRP) levels were significantly higher in the cPP group (2.59 mg/L (1.0–15.3)) compared to the cPA group (1.8 mg/L (0.7–4.5)), demonstrating a strong association with the presence of carotid plaques (P < 0.001). In contrast, triglyceride levels were similar between the groups (P = 0.50). However, dyslipidemia was significantly more common in the cPP group (59.4%) compared to the cPA group (47.9%) (P = 0.001). Chronic kidney disease (CKD) stages 4–5 (GFR < 30 mL/min/1.73 m²) were markedly more prevalent among cPP patients (19.8%) than in the cPA group (6.1%) (P < 0.001). A history of cardiovascular disease (CV disease) also revealed notable differences between the groups: heart failure was more prevalent in the cPP group (9.8% vs. 3.7%, P = 0.001), as was peripheral artery disease (19.6% vs. 10.5%, P < 0.001). Moreover, a history of gout flares was significantly more common in the cPP group compared to the cPA group (46.0% vs. 31.1%, P < 0.001). This finding suggests a potential link between gout flares and carotid plaque presence, underscoring the role of systemic inflammatory processes in the development of atherosclerosis. For a comprehensive overview of additional features, refer to Table 1.

**2.2 Variables Associated with the Presence of Carotid Plaques**

We conducted LASSO regression analysis to identify key variables associated with carotid plaque formation. The analysis revealed several non-zero coefficient variables, including age, BMI, uric acid levels, total cholesterol, low-density lipoprotein cholesterol (LDL-C), GFR < 60 mL/min, diabetes, coronary artery disease, history of stroke, antiplatelet therapy, urate-lowering therapy, antihypertensive medication use, Charlson comorbidity index, history and frequency of gout flares, as well as gout tophi and energy Doppler signals observed on musculoskeletal ultrasound. Each of these non-zero coefficient variables demonstrated a significant association with the presence of carotid plaques (Fig 1).

**References**

1.     Faul F, Erdfelder E, Buchner A, Lang A-G. Statistical power analyses using G*Power 3.1: tests for correlation and regression analyses. Behav Res Methods (2009) 41:1149–1160. doi: 10.3758/BRM.41.4.1149

2.     Touboul P-J, Hennerici MG, Meairs S, Adams H, Amarenco P, Bornstein N, et al. Mannheim carotid intima-media thickness and plaque consensus (2004-2006-2011). An update on behalf of the advisory board of the 3rd, 4th and 5th watching the risk symposia, at the 13th, 15th and 20th European Stroke Conferences, Mannheim, Germany, 2004, Brussels, Belgium, 2006, and Hamburg, Germany, 2011. Cerebrovasc Dis (2012) 34:290–296. doi: 10.1159/000343145

3.     Ibanez B, Fernández-Ortiz A, Fernández-Friera L, García-Lunar I, Andrés V, Fuster V. Progression of Early Subclinical Atherosclerosis (PESA) Study: JACC Focus Seminar 7/8. J Am Coll Cardiol (2021) 78:156–179. doi: 10.1016/j.jacc.2021.05.011

4.      Khan NF, Perera R, Harper S, Rose PW. Adaptation and validation of the Charlson Index for Read/OXMIS coded databases. BMC Fam Pract (2010) 11:1. doi: 10.1186/1471-2296-11-1

**Table 1:** Baseline Characteristics of Participants in Plaque and Non-Plaque Groups.

| Characteristics | cPA(N=409) | cPP(N=409) | P |
| --- | --- | --- | --- |
| Age in years, median (IQR) | 65.5 (60.0, 74.0) | 67.0 (60, 74.0) | 0.094 |
| Male, n (%) | 292 (71.4) | 292 (71.4) | 1.000 |
| BMI (kg/m^2^), median (IQR) | 24.4 (22.6, 26.7) | 24.5 (22.4, 26.6) | 0.275 |
| Obesity, n (%) | 59 (14.4) | 65 (15.9) | 0.626 |
| Alcohol, n (%) | 95 (23.2) | 103 (25.2) | 0.568 |
| Uric acid (umol/L), median (IQR) | 342.2 (256.8, 402.0) | 399.0 (341.2, 461.0) | < 0.001 |
| CRP (mg/L), median (IQR) | 1.8 (0.7, 4.5) | 2.6 (1.0, 15.3) | < 0.001 |
| Cholesterol (mmol/L), median (IQR) | 4.3 (3.5, 5.0) | 4.3 (3.4, 5.4) | 0.356 |
| Triglycerides (mmol/L), median (IQR) | 1.3 (0.9, 1.9) | 1.3 (1.0, 2.0) | 0.495 |
| HDL (mmol/L), median (IQR) | 1.1 (0.9, 1.4) | 1.1 (0.9, 1.3) | 0.432 |
| LDL (mmol/L), median (IQR) | 2.6 (2.0, 3.3) | 2.7(1.9, 3.6) | 0.684 |
| CKD (GFR<60 mL/min/1.73 m^2^),  n (%) | 25 (6.1) | 65 (15.9) | < 0.001 |
| CKD stage 4–5  (GFR < 30 mL/min/1.73 m^2^), n (%) | 25 (6.1) | 81 (19.8) | < 0.001 |
| Conventional risk factors present | |  |  |
| Smoker, n (%) | 112 (27.4) | 121 (29.6) | 0.535 |
| Hypertension, n (%) | 155 (37.9) | 285 (69.7) | < 0.001 |
| Dyslipidemia, n (%) | 196 (47.9) | 243 (59.4) | 0.001 |
| Diabetes, n (%) | 103 (25.2) | 212 (51.8) | < 0.001 |
| History of CV disease |  |  |  |
| Coronary heart disease, n (%) | 58 (14.2) | 106 (25.9) | < 0.001 |
| Heart failure, n (%) | 15 (3.7) | 40 (9.8) | 0.001 |
| Cerebrovascular disease, n (%) | 43 (10.5) | 116 (28.4) | < 0.001 |
| Peripheral artery disease, n (%) | 43 (10.5) | 80 (19.6) | 0.001 |
| Medications Taken |  |  |  |
| Statins, n (%) | 192 (49.6) | 281 (68.7) | < 0.001 |
| Antiplatelet drugs, n (%) | 109 (26.7) | 211 (51.6) | < 0.001 |
| Antihypertensive drugs, n (%) | 155 (37.9) | 288 (70.4) | < 0.001 |
| Urate-lowering therapy, n (%) | 108 (26.4) | 143 (35.0) | 0.010 |
| Latest urate-lowering drug prescribed | |  |  |
| Allopurinol, n (%) | 106 (98.6) | 138 (96.8) | 0.391 |
| Febuxostat, n (%) | 1 (0.9) | 1 (0.70) | 1.000 |
| Uricosurics†, n (%) | 0 (0.0) | 1 (0.70) | 1.000 |
| Gout flare, n (%) | 127 (31.1) | 188 (46.0) | < 0.001 |
| Charlson index, n (%) |  |  | < 0.001 |
| 0 | 67 (16.8) | 10 (2.4) |  |
| 1-2 | 202 (49.4) | 114 (27.9) |  |
| 3-5 | 98 (24.0) | 184 (45.0) |  |
| ≥6 | 18 (4.4) | 101 (24.7) |  |

†Uricosurics, including probenecid, benzoromarone, sulfinpyrazone

*The Charlson Comorbidity Index predicts mortality by weighting specific comorbidities (range, 0-29 [higher score indicates increased risk of mortality]) ^[4]^. Abbreviations: cPP, carotid plaque presence; cPA, carotid plaque absence; BMI, body mass index; CRP, C reactive protein; HDL-C, high density lipoprotein-cholesterol; LDL-C, low density lipoprotein-cholesterol; GFR, glomerular filtration rate.

**Figure 1**: LASSO Regression Results for Screening Risk Factors of Carotid Artery Plaque.

| 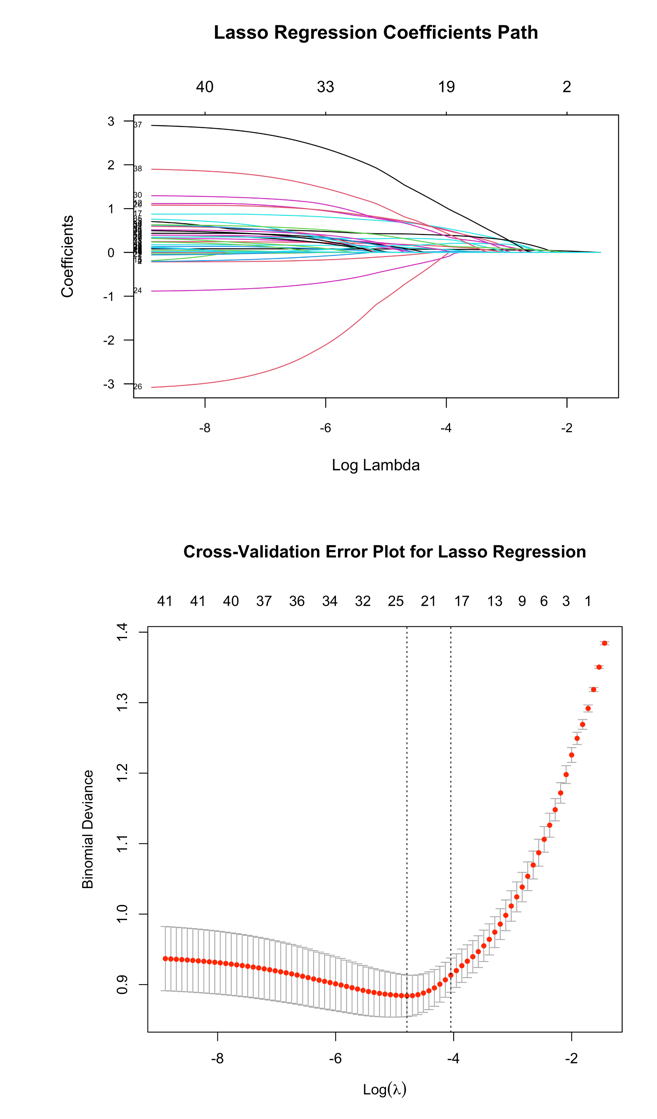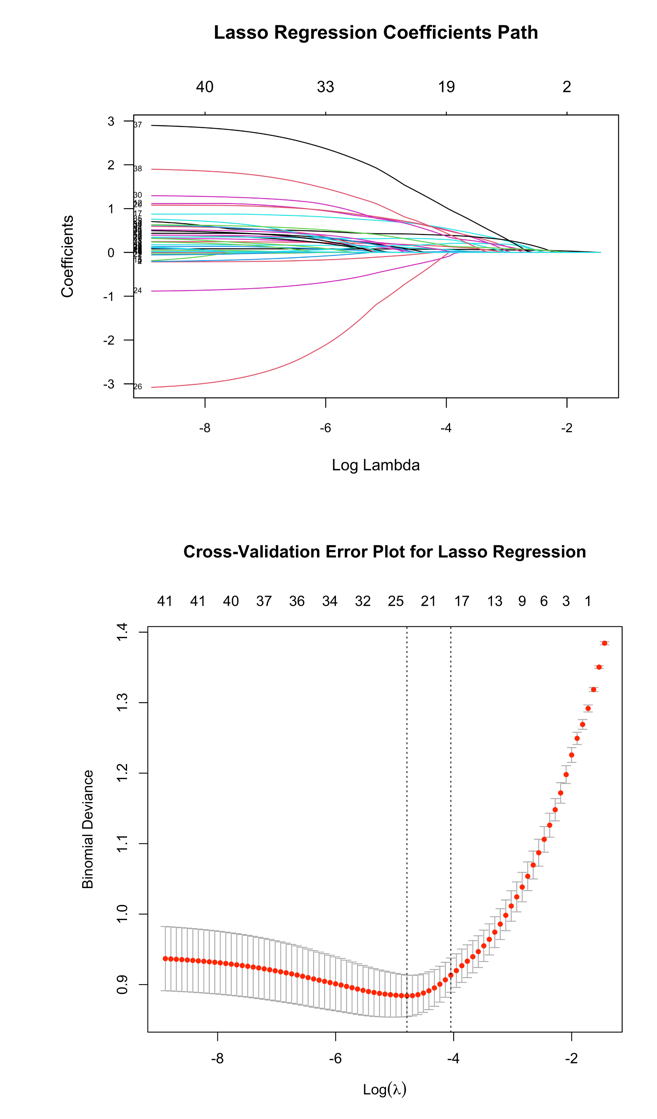 |
| --- |
