# Supplementary material for: Integrating ultrasound and clinical risk factors to predict carotid plaque vulnerability in gout patients: a machine learning approach
Source: Front Med (Lausanne). 2025 Jun 19;12:1556387. doi: 10.3389/fmed.2025.1556387 (PMC12224871; doi:10.3389/fmed.2025.1556387)
Supplement: Supplementary file 5 [file Data_Sheet_2.docx]

Supplementary Material

# Supplementary Data 2

# Secondary explanatory variables

Demographic characteristics

- Age (years)
- Gender
- Body mass index (BMI, kg/m^2^) at the time of diagnosis

Clinical characteristics

- Serum urate level (umol/L) at the time of diagnosis
- C-reactive protein (CRP, mg/L) at the time of diagnosis
- Cholesterol (mmol/L) at the time of diagnosis
- Triglycerides (mmol/L) at the time of diagnosis
- High-density lipoprotein level (HDL, mmol/L) at the time of diagnosis
- Low-density lipoprotein level (LDL, mmol/L) at the time of diagnosis
- Glomerular filtration rate (GFR, in mL/min/1.73m^2^, defined by CKD-EPI equation) at the time of diagnosis
- Chronic kidney disease (CKD): decreased estimated glomerular filtration rate (eGFR) < 60 mL/min/1.73 m² (in patients’ record)
- Chronic kidney disease stage 4-5 (if GFR <30 mL/min/1.73m^2^)
- Obesity (BMI ≥28)
- Alcohol (alcohol intake of ≥100 mL/day for more than one year)
- Smoker (by anamnesis)
- Hypertension: systolic blood pressure ≥ 140 mmHg and/or diastolic blood pressure ≥ 90 mmHg, or the use of antihypertensive medications (in patients’ record)
- Diabetes: fasting plasma glucose ≥ 126 mg/dL (7.0 mmol/L), 2-hour plasma glucose ≥ 200 mg/dL (11.1 mmol/L) during an oral glucose tolerance test, glycated hemoglobin (HbA1c) ≥ 6.5% (48 mmol/mol), or a prior clinical diagnosis of diabetes mellitus documented in patients’ record (in patients’ record)
- Dyslipidemia (clinical diagnosis in patients’ record)
- History of cardiovascular (CV) disease: coronary artery disease, heart failure, cerebrovascular disease and peripheral artery disease including aortic aneurism (in patients’ record)
- Medications taken include statins, antiplatelet drugs, antihypertensive drugs, and urate-lowering therapy (prescriptions confirmed within the year prior to cohort enrollment in patients’ record)
- Latest urate-lowering drug prescribed: Allopurinol Febuxostat or Uricosurics (including probenecid, benzoromarone, sulfinpyrazone) (in patients’ record)
- The Charlson Comorbidity Index predicts mortality by weighting specific comorbidities (range, 0-29 [higher score indicates increased risk of mortality]) (doi:10.1186/1471-2296-11-1)
