# Supplementary material for: Integrating ultrasound and clinical risk factors to predict carotid plaque vulnerability in gout patients: a machine learning approach
Source: Front Med (Lausanne). 2025 Jun 19;12:1556387. doi: 10.3389/fmed.2025.1556387 (PMC12224871; doi:10.3389/fmed.2025.1556387)
Supplement: Supplementary file 6 [file Data_Sheet_3.docx]

Supplementary Material

# Supplementary Data 3

# Comparison of key effect estimates across different models adjusting for alcohol and smoking.


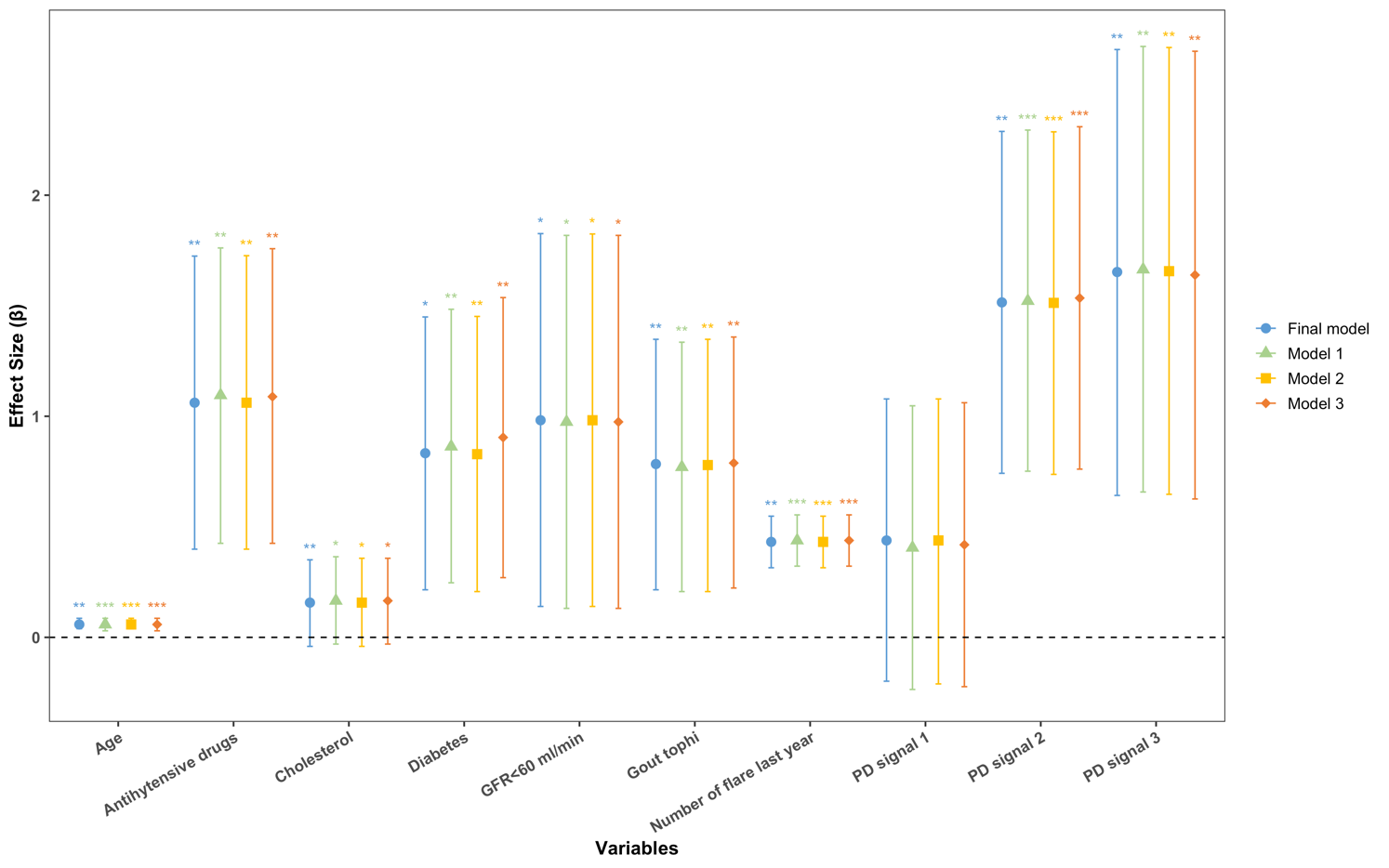


Supplementary Data 3: sensitivity analysis results indicated that the four models exhibited similar effect sizes (β), confidence intervals, and statistical significance across eight common variables, supporting the robustness of the main findings. The Final Model includes 8 primary variables selected after multivariable ordinal logistic regression analysis. Model 1 additionally incorporated alcohol consumption, Model 2 further included smoking, Model 3 simultaneously added adjustments for both alcohol and smoking. Significance levels are indicated as p < 0.05.
